# Comparing paired vs non-paired statistical methods of analyses when making inferences about absolute risk reductions in propensity-score matched samples

**DOI:** 10.1002/sim.4200

**Published:** 2011-02-21

**Authors:** Peter C Austin

**Affiliations:** a Institute for Clinical Evaluative SciencesToronto, Ont., Canada; b Department of Health Management, Policy and Evaluation, University of TorontoToronto, Ont., Canada; c Dalla Lana School of Public Health, University of TorontoToronto, Ont., Canada

**Keywords:** propensity score, propensity-score matching, risk difference, absolute risk reduction, Monte Carlo simulations, statistical inference, hypothesis testing, type I error rate, categorical data analysis

## Abstract

Propensity-score matching allows one to reduce the effects of treatment-selection bias or confounding when estimating the effects of treatments when using observational data. Some authors have suggested that methods of inference appropriate for independent samples can be used for assessing the statistical significance of treatment effects when using propensity-score matching. Indeed, many authors in the applied medical literature use methods for independent samples when making inferences about treatment effects using propensity-score matched samples. Dichotomous outcomes are common in healthcare research. In this study, we used Monte Carlo simulations to examine the effect on inferences about risk differences (or absolute risk reductions) when statistical methods for independent samples are used compared with when statistical methods for paired samples are used in propensity-score matched samples. We found that compared with using methods for independent samples, the use of methods for paired samples resulted in: (i) empirical type I error rates that were closer to the advertised rate; (ii) empirical coverage rates of 95 per cent confidence intervals that were closer to the advertised rate; (iii) narrower 95 per cent confidence intervals; and (iv) estimated standard errors that more closely reflected the sampling variability of the estimated risk difference. Differences between the empirical and advertised performance of methods for independent samples were greater when the treatment-selection process was stronger compared with when treatment-selection process was weaker. We recommend using statistical methods for paired samples when using propensity-score matched samples for making inferences on the effect of treatment on the reduction in the probability of an event occurring. Copyright © 2011 John Wiley & Sons, Ltd.

## 1. Introduction

Propensity-score matching is increasingly being used to estimate the effects of treatments, exposures and interventions on outcomes in observational studies. The propensity score is the probability of treatment assignment conditional on the observed baseline covariates 1. If treatment assignment is strongly ignorable, then conditioning on the propensity score allows one to obtain an unbiased estimate of average treatment effects 1.

Matching on the propensity score allows one to construct a matched sample in which systematic differences in observed baseline covariates are reduced or eliminated between treatment groups 2. Outcomes often can be directly compared between treatment groups in the propensity-score matched sample. There is a lack of consensus in the literature as to the appropriate statistical methods to use when assessing the statistical significance of the estimated treatment effect in a propensity-score matched sample. Schafer and Kang suggest that, within the matched sample, the treated and untreated subjects should be regarded as independent 3. Thus, if outcomes were continuous, a two-sample *t*-test could be used for testing the statistical significance of a difference in means between treatment groups, while the Pearson Chi-squared test could be used to test the statistical significance of the difference in proportions between treatment groups. Recent systematic reviews of propensity-score matching in the medical literature have found that authors frequently use statistical methods for independent samples when assessing the statistical significance of the estimated treatment effect in the propensity-score matched sample 4–6.

In contrast to this, it has been argued elsewhere that a propensity-score matched sample does not consist of independent observations 5. Treated and untreated subjects matched on the propensity score will have observed baseline covariates that come from the same multivariate distribution 1. Thus, on average, the baseline characteristics of matched treated and untreated subjects will be more similar than the baseline characteristics of randomly selected treated and untreated subjects in the propensity-score matched sample. When confounding is present, baseline covariates are related to the outcome. Therefore, the outcomes of matched treated and untreated subjects are likely to be more similar to one another compared to the outcomes of randomly selected treated and untreated subjects in the propensity-score matched sample.

In medical and clinical research, dichotomous outcomes are common 7, 8. Propensity score methods allow for direct estimation of risk differences (or absolute risk reductions), in which the proportion of subjects in whom the event occurs can be directly compared between treatment groups in the propensity-score matched sample 9. The objective of the current study was to compare the effect on statistical inference when statistical methods for independent samples are used compared with when statistical methods for paired samples are used for comparing differences in proportions between treatment groups in the propensity-score matched sample. The study had four specific objectives: First, to compare the empirical type I error rate when using McNemar's test compared with when using the standard Pearson Chi-squared test for comparing the proportion of subjects in whom the event occurred between treatment groups; second, to compare the empirical coverage rates of 95 per cent confidence intervals when standard errors are estimated using methods for paired data compared with when methods for independent samples are used; third, to compare the precision of estimated 95 per cent confidence intervals when standard errors are estimated using methods for paired data compared with when methods for independent samples are used; and fourth, to compare the variance of the empirical sampling distribution of the risk difference with the estimated variance of the risk difference when using methods for independent samples and methods for paired samples. These four objectives will be addressed using Monte Carlo simulations.

## 2. Monte Carlo simulations—methods

In this section, we describe the Monte Carlo simulations that were used to compare statistical inference when methods for independent samples were used compared with when methods for paired samples were used.

### 2.1. Data-generating process

We used a data-generating process identical to one used in a prior study that examined optimal caliper widths for use with propensity-score matching 10. Briefly, we simulated data sets such that approximately 25 per cent of the sample was exposed to the treatment. The data-generating process was designed to induce a specific average treatment effect for the treated (ATT), the measure of effect that is estimated when propensity-score matching is used 11. Furthermore, in the simulated data sets, the marginal probability of the outcome would be approximately 0.29 if all subjects in the population were not exposed. We then examined scenarios in which the risk differences due to treatment in treated subjects were 0, −0.02, −0.05, −0.10 and −0.15 (i.e. absolute reductions in the probability of the outcome due to treatment were 0, 0.02, 0.05, 0.10 and 0.15). First, we randomly generated 10 independent covariates (*X*_1_−*X*_10_) from independent standard normal distributions for each of 10 000 subjects. We then assumed that the following logistic regression model related the probability of treatment to these 10 baseline covariates: 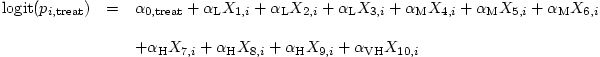 We then generated a treatment status indicator (*Z*_*i*_) for each subject from a Bernoulli distribution with subject-specific probability equal to *p*_*i*, treat_. Those subjects with *Z*_*i*_ = 1 denote the treated subjects in whom the ATT is defined. We assumed that the following logistic regression model related the probability of the outcome to these covariates and an indicator variable (*Z*) denoting treatment: 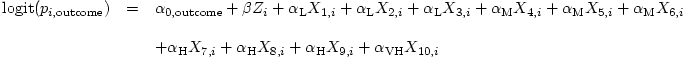 In the above regression model, *p*_*i*, outcome_ denotes the probability of the outcome for the *i*th subject and β denotes the log-odds ratio relating treatment to the outcome. We then generated subject-specific outcomes from a Bernoulli distribution with probability *p*_*i*, outcome_. The regression coefficients for the baseline covariates in the above two regression models were set as follows: α_L_ = log(1.1), α_M_ = log(1.25), α_H_ = log(1.5) and α_VH_ = log(2). These are intended to reflect low, medium, high and very high effect sizes. We fixed the value of α_0, treat_ = −1.344090 so that approximately 25 per cent of the subjects would be treated. We fixed the value of α_0, outcome_ = −1.098537 so that the probability of the event occurring in the population if all subjects were untreated would be approximately 0.29. To induce a risk difference of 0, β was set to be 0. For risk differences of −0.02, −0.05, −0.10 and −0.15, the required value of β equaled log(0.90619), log(0.7795362), log(0.6001387) and log(0.45292), respectively. The reader is referred elsewhere for a more detailed explanation of how these values of β were determined 12.

The above scenario assumed that the 10 covariates (*X*_1_−*X*_10_) were all independently distributed standard normal random variables. We also examined four additional covariate scenarios. In the second covariate scenario, the 10 covariates were from a multivariate normal distribution such that the mean and variance of each random variable were equal to 0 and 1, respectively, while the correlation between pairs of random variables was equal to 0.25. In the third covariate scenario, the first five covariates (*X*_1_−*X*_5_) were assumed to be independent Bernoulli random variables with parameter 0.5, while the last five covariates (*X*_6_−*X*_10_) were assumed to be independent standard normal random variables. In the fourth covariate scenario, the first nine covariates were assumed to be independent Bernoulli random variables with parameter 0.5, while the tenth covariate was a standard normal random variable. In the fifth covariate scenario, all the 10 covariates (*X*_1_−*X*_10_) were all independent Bernoulli random variables with parameter 0.5. The values of α_0, treat_, α_0, outcome_ and β were modified in order to preserve the proportion of treated subjects, the marginal probability of the outcome, and the required treatment effect. We refer to the five covariate scenarios as the independent normal covariates scenario, the correlated normal covariates scenario, the first mixed covariates scenario, the second mixed covariates and the independent Bernoulli covariates scenario, respectively. Within each of the five covariate scenarios and for each of the five absolute risk reductions, we randomly generated 1825 data sets, each consisting of 10 000 subjects. We refer to the above set of 25 scenarios as the scenarios with a 0.29 outcome probability and a weak treatment-selection model.

We also examined three additional sets of five covariate scenarios. In the next set of five scenarios, the data-generating process was modified so that the probability of the outcome if all subjects were untreated was 0.15. We refer to this set of five 25 scenarios as the scenarios with a 0.15 outcome probability and a weak treatment-selection model. We then modified these two sets of 25 scenarios by changing the weak treatment-selection model to a strong treatment-selection model. A strong treatment-selection process will induce greater differences in baseline covariates between treated and untreated subjects in the unmatched sample. In these two sets of 25 scenarios, the coefficients for the treatment-selection model and the outcomes model were changed to: α_L_ = log(1.5), α_M_ = log(1.75), α_H_ = log(2), and α_VH_ = log(2.5). In the two sets of simulations in which there was a strong treatment-selection model, we observed low percentages of treated subjects successfully matched to untreated subjects in some of the covariate scenarios. Therefore, in these two sets of scenarios, minor modifications were made to the data-generating process by adding additional untreated subjects to the sample. Ten additional copies of each untreated subject were created within each replication of the Monte Carlo simulations. For these 10 additional subjects, outcomes were generated independently using the same subject-specific probability of an outcome. In these last two sets of simulations, the initially generated data set was of size 1000 (rather than of size 10 000). Then 10 copies of each untreated subject were added to the simulated sample so as to increase the number of potential control subjects.

### 2.2. Statistical analyses

In each simulated data set, we estimated the propensity score using a logistic regression model to regress treatment status on the 10 baseline covariates. Propensity-score matching was used to construct a matched sample consisting of pairs of treated and untreated subjects. We used greedy nearest neighbor matching on the logit of the propensity score using a caliper of width equal to 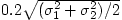, where 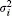 is the variance of the logit of the propensity score in the *i*th treatment group. This caliper width was used as it has been shown to result in optimal estimation of risk differences in a variety of settings 10.

In the propensity-score matched sample, the absolute risk reduction was estimated as the sample difference of the proportion of treated subjects in whom the outcome occurred and the proportion of untreated subjects in whom the outcome occurred in the propensity-score matched sample. When the true absolute risk reduction was 0 (the null hypothesis), the statistical significance of the estimated risk difference was assessed using two different methods. First, using methods for independent samples, the Pearson Chi-squared was used to assess the statistical significance of the difference in the probability of the outcome occurring between treatment groups 13. Second, using methods for paired samples, McNemar's test was used for this comparison.

The variance of the difference in proportions was estimated in two different methods. First, using methods for independent samples, let *p*_*T*_ and *p*_*C*_ denote the observed probability of the outcome occurring in treated and untreated subjects, respectively, in the propensity-score matched sample. Furthermore, assume that there are *N* propensity score matched pairs. Then the standard error of the estimated risk difference is given by 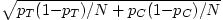 13. Second, using methods for paired samples, we assume that in the matched sample there were *a* pairs in which both the treated and untreated subjects experienced the event; *b* pairs in which the treated subject experienced the event while the untreated subject does not; and *c* pairs in which the untreated subject experienced the event while the treated subject did not. Then, the variance of the difference in proportions was estimated by ((*b*+ *c*)−(*c*−*b*)^2^/*n*)/*n*^2^^14^. In both cases, 95 per cent confidence intervals were estimated as *p*_*T*_−*p*_*C*_±1.96 × se(*p*_*T*_−*p*_*C*_), where se(*p*_*T*_−*p*_*C*_) denotes the estimated standard error of the risk difference.

For each of the 100 scenarios (2 treatment−selection models × 2 probabilities of outcomes × 5covariate scenarios × 5 absolute risk reductions), we simulated 1825 data sets. The above analyses were conducted using each of the 1825 simulated data sets. In the 20 scenarios in which the true risk difference was 0, we estimated the empirical type I error rate as the proportion of simulated data sets in which the null hypothesis of no-treatment effect was rejected with a significance level of less than 0.05. Owing to our use of 1825 simulated data sets, an empirical type I error rate that was less than 0.04 or greater than 0.06 would be classified as being statistically significantly different from 0.05. For each of the 100 scenarios, we determined the proportion of estimated 95 per cent confidence intervals that contained the true risk difference. As above, due to the use of 1825 simulated data sets, empirical coverage rates that are less than 0.94 or that exceed 0.96 are statistically significantly different from the advertised coverage rate of 0.95. We also determined the mean width of the estimated 95 per cent confidence intervals across the 1825 simulated data sets. Finally, we compared the standard deviation of the empirical sampling distribution of the estimated treatment effects (i.e. the standard deviation of the 1825 estimated risk differences across the simulated data sets) with the mean of the estimated standard errors of the estimated treatment effect.

## 3. Monte Carlo simulations—results

The empirical type I error rates for the 20 different scenarios in which there was a true null treatment effect are reported in Table I. We also report the mean percentage of treated subjects that were successfully matched to an untreated subject across the 1825 simulated samples. When the Pearson Chi-squared test was used to test the statistical significance of the risk difference, the empirical type I error rates were less than 0.04 in 14 (70 per cent) of the 20 different scenarios. In contrast, when McNemar's test was used, the empirical type I error rate were never less than 0.04. In one (5 per cent) of the 20 scenarios, the empirical type I error rate exceeded 0.06 (the empirical type I error rate was 0.0614 in this scenario). Thus, in the majority of covariate scenarios, the use of a method for independent samples (i.e. the Chi-squared test) resulted in a type I error rate that was statistically significantly different from the advertised rejection rate. When the results were stratified by the strength of the treatment-selection process, when methods for independent samples were used, the empirical type I error rate was statistically significantly different from 0.05 in 40 per cent of the 10 scenarios when there was a weak treatment-selection process; however, the empirical type I error rate was statistically significantly different from 0.05 in 100 per cent of the scenarios when there was a strong treatment-selection process.

**Table I:** Empirical type I error rates for statistical methods for paired vs independent samples

Covariate scenario	Mean percent of treated subjects matched	Pearson chi-squared test (independent sample method)	McNemar's test (paired sample method)
*0.29 outcome probability, weak treatment-selection model*			
Independent normal	90.4	0.0427	0.0614
Correlated normal	75.2	0.0351	0.0537
Mixture scenario 1	91.4	0.0296	0.0521
Mixture scenario 2	96.1	0.0422	0.0499
Independent Bernoulli	99.7	0.0416	0.0477
*0.15 outcome probability, weak treatment-selection model*			
Independent normal	90.4	0.0378	0.0493
Correlated normal	75.2	0.0345	0.0537
Mixture scenario 1	91.4	0.0405	0.0499
Mixture scenario 2	96.1	0.0427	0.0515
Independent Bernoulli	99.7	0.051	0.0537
*0.29 outcome probability, strong treatment-selection model*			
Independent normal	93.5	0.0208	0.0405
Correlated normal	78.6	0.0208	0.0526
Mixture scenario 1	95.2	0.029	0.0526
Mixture scenario 2	97.7	0.0334	0.051
Independent Bernoulli	99	0.0395	0.0532
*0.15 outcome probability, strong treatment-selection model*			
Independent normal	93.5	0.0258	0.0521
Correlated normal	78.6	0.0214	0.0466
Mixture scenario 1	95.2	0.0323	0.0477
Mixture scenario 2	97.7	0.0312	0.0433
Independent Bernoulli	99	0.0384	0.0504

*Note*: Cells contain results averaged over 1825 Monte Carlo simulations.

The empirical coverage rates of 95 per cent confidence intervals, the mean length of 95 per cent confidence intervals, and the ratio of the mean length of estimated 95 per cent confidence intervals obtained using methods for independent samples to the mean length of estimated 95 per cent confidence intervals obtained using methods for paired samples are reported in Table II–V for the four different sets of five covariate scenarios. In 71 of the 100 scenarios, the empirical coverage rates of 95 per cent confidence intervals obtained using a method for independent samples were statistically significantly different from 0.95 (i.e. empirical coverage rates exceeded 0.96 or were less than 0.94). The median empirical coverage rate was 0.964 (25th and 75th percentiles: 0.955 and 0.971) across the 100 scenarios. Thus, in over half of the 100 scenarios, the empirical coverage rates were significantly different from the advertised rate of 0.95. In contrast, in 15 of the 100 scenarios, the empirical coverage rates of 95 per cent confidence intervals obtained using methods for paired samples were statistically significantly different from 0.95. The median empirical coverage rate was 0.949 (25th and 75th percentiles: 0.944 and 0.951). As above, we examined the results for the independent method of analysis separately in scenarios with a weak treatment selection-process and in scenarios with a strong treatment selection-process. In 24 (48 per cent) of the 50 scenarios with a weak treatment-selection process, methods for independent samples resulted in 95 per cent confidence intervals whose empirical coverage rates were not statistically significantly different from 0.95. However, in only 5 (10 per cent) of the 50 scenarios with a strong treatment-selection process, did methods for independent samples result in 95 per cent confidence intervals whose empirical coverage rates were not statistically significantly different from 0.95.

**Table II:** Coverage and width of empirical 95 per cent confidence intervals and estimation of sampling variances of treatment effects −0.29 treatment effect, weak treatment-selection model

	Coverage of 95 per cent confidence intervals		Lengths of 95 per cent confidence intervals		Ratio of length of independent CI to paired CI	Ratio of mean estimated variance of treatment effect to variance of empirical sampling distribution	
				
True risk difference	Independent	Paired	Independent	Paired		Independent	Paired
*Independent normal covariates*							
0	0.957	0.938	0.057	0.053	1.075	1.084	0.944
−0.02	0.963	0.947	0.057	0.053	1.075	1.076	0.938
−0.05	0.964	0.949	0.056	0.052	1.077	1.093	0.956
−0.1	0.963	0.95	0.055	0.051	1.078	1.126	0.99
−0.15	0.961	0.944	0.053	0.05	1.06	1.138	1.008
*Correlated normal covariates*							
0	0.965	0.946	0.063	0.057	1.105	1.255	1.02
−0.02	0.975	0.958	0.063	0.057	1.105	1.247	1.016
−0.05	0.97	0.948	0.063	0.057	1.105	1.24	1.013
−0.1	0.972	0.951	0.062	0.056	1.107	1.262	1.037
−0.15	0.969	0.952	0.061	0.055	1.109	1.244	1.032
*Mixture covariate scenario 1*							
0	0.97	0.948	0.056	0.053	1.057	1.131	0.991
−0.02	0.964	0.951	0.056	0.053	1.057	1.124	0.986
−0.05	0.961	0.95	0.056	0.052	1.077	1.111	0.977
−0.1	0.964	0.95	0.054	0.051	1.059	1.122	0.992
−0.15	0.962	0.949	0.053	0.05	1.06	1.124	1.001
*Mixture covariate scenario 2*							
0	0.958	0.95	0.054	0.052	1.038	1.091	0.988
−0.02	0.958	0.95	0.054	0.051	1.059	1.093	0.991
−0.05	0.962	0.951	0.053	0.051	1.039	1.07	0.972
−0.1	0.953	0.942	0.052	0.05	1.04	1.07	0.977
−0.15	0.955	0.945	0.05	0.048	1.042	1.085	0.998
*Independent Bernoulli covariates*							
0	0.958	0.952	0.052	0.051	1.02	1.07	1.014
−0.02	0.953	0.95	0.052	0.05	1.04	1.078	1.023
−0.05	0.955	0.95	0.051	0.05	1.02	1.077	1.024
−0.1	0.95	0.946	0.05	0.048	1.042	1.072	1.022
−0.15	0.959	0.954	0.048	0.047	1.021	1.058	1.014

**Table III:** Coverage and width of empirical 95 per cent confidence intervals and estimation of sampling variances of treatment effects −0.15 treatment effect, weak treatment-selection model

	Coverage of 95 per cent confidence intervals		Lengths of 95 per cent confidence intervals		Ratio of length of independent CI to paired CI	Ratio of mean estimated variance of treatment effect to variance of empirical sampling distribution	
				
True risk difference	Independent	Paired	Independent	Paired		Independent	Paired
*Independent normal covariates*							
0	0.962	0.951	0.047	0.045	1.044	1.113	1.005
−0.02	0.953	0.942	0.047	0.044	1.068	1.097	0.993
−0.05	0.944	0.929	0.045	0.043	1.047	1.07	0.974
−0.1	0.873	0.856	0.043	0.041	1.049	1.074	0.99
−0.15	0.685	0.665	0.04	0.039	1.026	1.073	1.009
*Correlated normal covariates*							
0	0.965	0.946	0.053	0.049	1.082	1.193	1.019
−0.02	0.956	0.939	0.053	0.049	1.082	1.207	1.034
−0.05	0.923	0.895	0.051	0.048	1.063	1.192	1.027
−0.1	0.722	0.676	0.049	0.046	1.065	1.153	1.005
−0.15	0.311	0.265	0.047	0.044	1.068	1.138	1.01
*Mixture covariate scenario 1*							
0	0.959	0.95	0.047	0.045	1.044	1.105	1.004
−0.02	0.956	0.944	0.046	0.044	1.045	1.106	1.008
−0.05	0.953	0.944	0.045	0.043	1.047	1.126	1.03
−0.1	0.91	0.896	0.042	0.041	1.024	1.099	1.018
−0.15	0.744	0.723	0.039	0.038	1.026	1.058	0.998
*Mixture covariate scenario 2*							
0	0.957	0.948	0.045	0.043	1.047	1.039	0.968
−0.02	0.952	0.944	0.044	0.042	1.048	1.027	0.959
−0.05	0.95	0.942	0.042	0.041	1.024	0.994	0.932
−0.1	0.925	0.919	0.04	0.039	1.026	0.955	0.906
−0.15	0.883	0.877	0.036	0.036	1	0.95	0.917
*Independent Bernoulli covariates*							
0	0.949	0.946	0.042	0.042	1	1.038	1.002
−0.02	0.955	0.949	0.041	0.041	1	1.03	0.996
−0.05	0.952	0.95	0.04	0.039	1.026	1.031	1.001
−0.1	0.953	0.951	0.036	0.036	1	0.983	0.961
−0.15	0.946	0.945	0.032	0.032	1	0.972	0.962

**Table IV:** Coverage and width of empirical 95 per cent confidence intervals and estimation of sampling variances of treatment effects −0.29 treatment effect, strong treatment-selection model

	Coverage of 95 per cent confidence intervals		Lengths of 95 per cent confidence intervals		Ratio of length of independent CI to paired CI	Ratio of mean estimated variance of treatment effect to variance of empirical sampling distribution	
				
True risk difference	Independent	Paired	Independent	Paired		Independent	Paired
*Independent normal covariates*							
0	0.979	0.958	0.181	0.157	1.153	1.391	1.044
−0.02	0.980	0.956	0.181	0.157	1.153	1.391	1.044
−0.05	0.976	0.951	0.181	0.157	1.153	1.370	1.032
−0.1	0.975	0.950	0.180	0.157	1.146	1.356	1.026
−0.15	0.979	0.957	0.179	0.156	1.147	1.398	1.066
*Correlated normal covariates*							
0	0.979	0.944	0.188	0.157	1.197	1.470	1.027
−0.02	0.982	0.945	0.189	0.158	1.196	1.478	1.034
−0.05	0.981	0.952	0.191	0.160	1.194	1.508	1.057
−0.1	0.968	0.928	0.193	0.162	1.191	1.470	1.039
−0.15	0.947	0.897	0.193	0.163	1.184	1.334	0.954
*Mixture covariate scenario 1*							
0	0.970	0.946	0.180	0.158	1.139	1.251	0.966
−0.02	0.976	0.947	0.180	0.158	1.139	1.288	0.995
−0.05	0.971	0.942	0.179	0.158	1.133	1.240	0.961
−0.1	0.969	0.945	0.178	0.157	1.134	1.268	0.987
−0.15	0.964	0.940	0.176	0.156	1.128	1.218	0.956
*Mixture covariate scenario 2*							
0	0.966	0.949	0.176	0.161	1.093	1.205	1.003
−0.02	0.970	0.951	0.176	0.160	1.100	1.206	1.004
−0.05	0.969	0.944	0.175	0.159	1.101	1.207	1.005
−0.1	0.968	0.951	0.172	0.157	1.096	1.223	1.021
−0.15	0.968	0.946	0.169	0.155	1.090	1.191	1.003
*Independent Bernoulli covariates*							
0	0.959	0.946	0.172	0.161	1.068	1.137	0.989
−0.02	0.964	0.948	0.172	0.160	1.075	1.117	0.973
−0.05	0.966	0.948	0.170	0.159	1.069	1.150	1.004
−0.1	0.965	0.951	0.167	0.156	1.071	1.128	0.990
−0.15	0.966	0.956	0.163	0.153	1.065	1.121	0.992

**Table V:** Coverage and width of empirical 95 per cent confidence intervals and estimation of sampling variances of treatment effects −0.15 treatment effect, strong treatment-selection model

	Coverage of 95 per cent confidence intervals		Lengths of 95 per cent confidence intervals		Ratio of length of independent CI to paired CI	Ratio of mean estimated variance of treatment effect to variance of empirical sampling distribution	
				
True risk difference	Independent	Paired	Independent	Paired		Independent	Paired
*Independent normal covariates*							
0	0.974	0.947	0.168	0.148	1.135	1.252	0.976
−0.02	0.974	0.952	0.167	0.147	1.136	1.286	1.006
−0.05	0.972	0.946	0.164	0.145	1.131	1.257	0.989
−0.1	0.974	0.952	0.159	0.142	1.120	1.265	1.012
−0.15	0.968	0.948	0.152	0.138	1.101	1.199	0.984
*Correlated normal covariates*							
0	0.978	0.953	0.188	0.161	1.168	1.360	0.997
−0.02	0.977	0.950	0.187	0.160	1.169	1.341	0.988
−0.05	0.973	0.945	0.184	0.159	1.157	1.297	0.963
−0.1	0.970	0.945	0.179	0.156	1.147	1.262	0.954
−0.15	0.973	0.949	0.173	0.152	1.138	1.259	0.977
*Mixture covariate scenario 1*							
0	0.967	0.952	0.164	0.147	1.116	1.234	0.993
−0.02	0.972	0.953	0.162	0.146	1.110	1.236	0.997
−0.05	0.973	0.951	0.159	0.144	1.104	1.296	1.055
−0.1	0.971	0.955	0.154	0.139	1.108	1.248	1.030
−0.15	0.969	0.948	0.147	0.135	1.089	1.208	1.020
*Mixture covariate scenario 2*							
0	0.969	0.956	0.154	0.143	1.077	1.208	1.042
−0.02	0.973	0.957	0.152	0.141	1.078	1.228	1.061
−0.05	0.973	0.957	0.148	0.138	1.072	1.234	1.071
−0.1	0.965	0.955	0.141	0.133	1.060	1.188	1.047
−0.15	0.961	0.950	0.133	0.126	1.056	1.151	1.039
*Independent Bernoulli covariates*							
0	0.960	0.950	0.146	0.139	1.050	1.117	1.008
−0.02	0.964	0.954	0.143	0.136	1.051	1.148	1.039
−0.05	0.963	0.954	0.139	0.133	1.045	1.140	1.037
−0.1	0.957	0.950	0.131	0.126	1.040	1.079	0.996
−0.15	0.948	0.939	0.122	0.118	1.034	1.004	0.947

The ratio between the mean length of confidence intervals obtained using methods for independent samples and the mean length of confidence intervals obtained using methods for paired samples ranged from a low of 1 to a high of 1.197; the median ratio was 1.074 (25th and 75th percentiles: 1.045 and 1.113) across the 100 scenarios. Thus, in half of the 100 scenarios, the estimated confidence intervals were at least 7.4 per cent wider when methods for independent samples were used compared with when methods for paired samples were used. As above, the relative difference between the widths of the confidence intervals was greater when there was a strong treatment-selection process compared with when there was a weak treatment-selection process.

The analyses reported in the above two paragraphs suggest that confidence intervals constructed using methods for paired samples tend to have coverage rates that were closer to the advertised rates compared with when methods for independent samples were used. Furthermore, methods for paired samples resulted in estimates with greater precision, since the estimated confidence intervals are narrower compared with when methods for independent samples were used.

The square of the ratio between the mean estimated standard error when methods for independent samples were used and the standard deviation of the empirical sampling distribution of the estimated risk differences across the 1825 simulated data sets is reported in the second rightmost column of Table II–V. A similar ratio obtained when methods for paired samples was used is reported in the rightmost column of Table II–V. When methods for independent samples were used, this ratio ranged from a low of 0.950 to a high of 1.508; the median ratio was 1.149 (25th and 75th percentiles: 1.085 and 1.250, respectively) across the 100 scenarios. Thus, in 50 per cent of the scenarios, variance estimates obtained using methods for independent samples overestimated the sampling variance of the estimated risk difference by at least 14.9 per cent. In 25 per cent of the scenarios, these methods overestimated the sampling variance of the estimated risk difference by at least 25.0 per cent. Furthermore, the estimated standard error overestimated the empirical standard deviation of the sampling distribution to a greater extent when there was a strong treatment-selection process compared with when there was a weak treatment-selection process. When methods for paired samples were used, the ratio ranged from a low of 0.906 to a high of 1.071; the median ratio was 1.003 (25th and 75th percentiles: 0.985 and 1.023, respectively) across the 100 scenarios.

## 4. Discussion

We compared statistical inference when methods for independent samples were used compared with when methods for paired samples were used for significance testing and for variance estimation when estimating risk differences in propensity-score matched samples. We found that compared with using methods for independent samples, the use of methods for paired samples resulted in: (i) empirical type I error rates that were closer to the advertised rate; (ii) empirical coverage rates of 95 per cent confidence intervals that were closer to the advertised rate; (iii) narrower 95 per cent confidence intervals; and (iv) estimated standard errors that were more closely reflected the sampling variability of the estimated risk difference.

As noted in the Introduction, applied researchers using propensity-score matching frequently use statistical methods for independent samples when estimating the statistical significance of estimated treatment effects 5. The results of our series of Monte Carlo simulations suggest than when outcomes are dichotomous and the risk difference (or the absolute risk reduction) is used as the measure of treatment effect, then statistical methods of inference that account for the matched nature of the propensity-score matched sample are preferable to methods for the analysis of independent samples. The use of methods for independent samples will result in conservative confidence intervals – that is, confidence intervals whose coverage rates exceed the advertised rate. Furthermore, the estimated confidence intervals will tend to be wider, with an associated loss in precision, when methods for independent samples are used compared with when methods for matched samples are used. However, one should note that the estimated risk difference does not depend on whether one assumes that methods for matched samples or methods for independent samples should be used.

The current study complements prior published research. An earlier study found that in many settings, methods for paired samples tended to result in improved inference compared with when methods for independent samples were used for the analysis of propensity-score matched samples 15. In the prior study, empirical coverage rates of confidence intervals and variance estimation was studied for differences in means, rate ratios and relative risks. Furthermore, empirical type I error rates were studied for these three measures of effects as well as for odds ratios and hazard ratios. This prior study did not examine inferences about risk differences. The recent description of a data-generating process for simulating data in which treatment induces a specified absolute risk reduction 12 permitted the examination of inferences for risk differences that was conducted in the current study.

It is important to examine the effect of different methods of analysis on inference when estimating risk differences or absolute risk reductions. Binary outcomes are common in healthcare research 8. When outcomes are binary, the effect of treatment on outcomes can be described using four different metrics: the risk difference, the relative risk, the number needed to treat (NNT) and the odds ratio. Propensity-score matching has been shown to result in biased estimation of both conditional and marginal odds ratios 16, 17. The NNT is the reciprocal of the absolute risk reduction. Comparisons of different propensity score methods for estimating absolute risk reductions and relative risks are described in greater detail elsewhere 9, 18.

In conclusion, we recommend that when propensity-score matching is used to reduce or eliminate the effects of treatment selection bias or confounding, that statistical methods for paired samples be used when estimating the effect of treatment or exposure on absolute risk reductions or risk differences.
